# Enzymatic Hydrolysis of Marine Collagen and Fibrinogen Proteins in the Presence of Thrombin

**DOI:** 10.3390/md18040208

**Published:** 2020-04-11

**Authors:** Ludmila L. Semenycheva, Marfa N. Egorikhina, Victoria O. Chasova, Natalya B. Valetova, Yulia L. Kuznetsova, Alexander V. Mitin

**Affiliations:** 1 Faculty of Chemistry, Lobachevsky State University of Nizhny Novgorod, pr. Gagarina 23, 603950 Nizhny Novgorod, Russia; 11.04.96@mail.ru (V.O.C.); nata-bor-2005@mail.ru (N.B.V.); kyul@yandex.ru (Y.L.K.); ckp@ichem.unn.ru (A.V.M.); 2 Federal State Budgetary Educational Institution of Higher Education Privolzhsky Research Medical University of the Ministry of Health of the Russian Federation, Minin and Pozharsky square 10/1, 603950 Nizhny Novgorod, Russia; egorihina.marfa@yandex.ru

**Keywords:** scaffold, biopolymers, fibrinogen, fibrin, collagen, hydrolysis, thrombin

## Abstract

Enzymatic hydrolysis of native collagen and fibrinogen was carried out under comparable conditions at room temperature. The molecular weight parameters of proteins before and after hydrolysis by thrombin were monitored by gel-penetrating chromatography (GPC). An analysis of the experiment results shows that the molecular weight parameters of the initial fibrinogen (Fn) and cod collagen (CC) are very similar. High molecular CC decays within the first minute, forming two low molecular fractions. The main part (~80%) falls on the fraction with a value of M_w_ less than 10 kDa. The initial high molecular fraction of Fn with M_w_ ~320–340 kDa is not completely hydrolyzed even after three days of control. The presence of low molecular fractions with M_w_ ~17 and M_w_ ~10 kDa in the solution slightly increases within an hour and noticeably increases for three days. The destruction of macromolecules of high molecular collagen to hydrolysis products appears almost completely within the first minute mainly to the polymer with M_w_ ~10 kDa, and enzymatic hydrolysis of fibrinogen proceeds slower than that of collagen, but also mainly to the polymer with M_w_ ~10 kDa. Comparative photos of the surfaces of native collagen, fibrinogen and the scaffold based on them were obtained.

## 1. Introduction

Scaffold technology is the most dynamically developing direction in tissue engineering. Fibrinogen/fibrin and collagen are among the most popular natural polymers for the formation of hydrogel scaffolds [1,2]. These proteins have a number of unique properties that make it possible to create a three-dimensional structure with a biomimetic architecture and function [3,4,5,6]. Scaffolds formed on the basis of fibrinogen and collagen can have a high degree of hydration, porosity, and a microfiber structure, which provides an extensive surface area for cells’ attachment and conditions for maintaining their viability, migration, and proliferation [7,8]. The demand for these proteins in tissue engineering is, to a large extent, related to their biological activity. They have open centers of interaction with cells’ integrins, which remain accessible after polymerization [9,10]. Due to the process of binding with cells’ integrins, fibrinogen/fibrin and collagen are able to activate intracellular signaling paths and, thus, regulate the tissue development process [11].

Comparatively, not long ago, marine collagen isolated from fish scales, skin, and bones was used in scaffold technology. A structural analysis of the protein confirmed that fish have collagen type I similar to collagen type I of mammals and birds [12,13]. Marine collagen is becoming more and more popular in tissue engineering and biomedical research due to several advantages over mammalian collagen: high biocompatibility, biodegradability, easy extractability, solubility in water, safety in relation to disease transfer, low immunogenicity and low production costs [14,15,16].

A number of methods of scaffold formation based on these biopolymers have been carried out in the presence of the proteolytic enzyme—thrombin, hydrolyzing peptide bonds [17,18]. Newly formed fragments of protein macromolecules form new bonds. As a result, macromolecules appear that differ from the original architecture [19,20,21,22]. This is the scaffold that represents hydrogel with certain characteristics of elasticity, transparency and density [23].

**Scheme 1:**
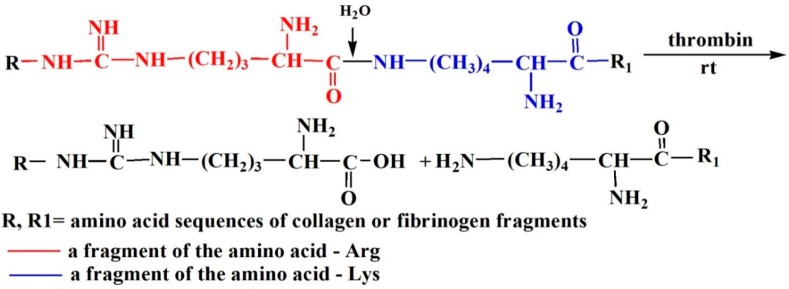
Hydrolysis of the Arg-–Lys bond in a protein molecule by thrombin.

It is not entirely clear how collagen interacts with fibrin during the formation of composite scaffolds under enzymatic hydrolysis conditions, however it is well known that thrombin hydrolyzes the bonds formed by arginine and lysine (Scheme 1) [24].

It was found earlier that the hydrolysis of native high molecular collagen of type I, isolated from the skin of different animals, under standard conditions in the presence of the enzyme—pancreatin proceeds at a sufficiently high rate [25].

Due to the fact that both proteins-collagen and fibrinogen/fibrin- are structure-forming proteins in fibrin-collagen scaffolds, the purpose of this work is to analyze the hydrolysis rate of high molecular cod collagen (CC) and fibrinogen (Fn) isolated from blood plasma in the presence of the enzyme—thrombin (used in the scaffold formation [1,17,18,26]). This is done by comparing the molecular weight parameters of protein hydrolysis products, as well as the structure analysis of the initial proteins and the scaffold formed on their basis.

## 2. Results

Enzymatic hydrolysis of collagen and fibrinogen was performed under comparable conditions. The molecular weight parameters of proteins in the process of enzymatic hydrolysis by thrombin are presented in Table 1.

The analysis of the molecular weight values shows that the values of the M_w_ fraction, the share of which is the largest in the initial samples of Fn and CC, differ: Fn has a slightly higher value. It can be noted that in the initial CC there are two fractions with values, mainly M_w_ ~300 kDa, which corresponds to the literature data [27], and M_w_ ~18 kDa. The appearance of the latter fraction is associated with the partial hydrolysis of collagen in the process of extraction from the natural substrate [28]. There are three such fractions in the case of Fn: values mainly M_w_ ~300 kDa, which corresponds to the literature data [29], M_w_ ~17 kDa and M_w_ ~10 kDa. The presence of two low molecular fibrinogen fractions is explained by the partial hydrolysis of the protein. Low molecular fractions are preserved during hydrolysis for three days (Table 1, lines 2, 3, 4, 5). As can be seen from the table data, after the first minute of hydrolysis, the initial CC decays to form two low molecular fractions. The main part (~80%) falls on the fraction with a value of M_w_ less than 10 kDa. This preserves a small amount of high molecular collagen in the solution. The ratio of fractions does not change at further control within three days.

Other regularities are observed for Fn. The initial high molecular fraction with M_w_ ~320–340 kDa exists in the solution during the entire hydrolysis period; its content varies slightly within an hour. After three days, ~16% of this fraction remains in the solution. The content in the solution of low molecular fractions with M_w_ ~17 kDa and M_w_ ~10 kDa slightly increases within an hour, but noticeably increases within three days. Probably, fibrin-polymer is formed in the system, but under the conditions of the analysis given in this article, such a molecular weight is not identified.

**Figure 1:**
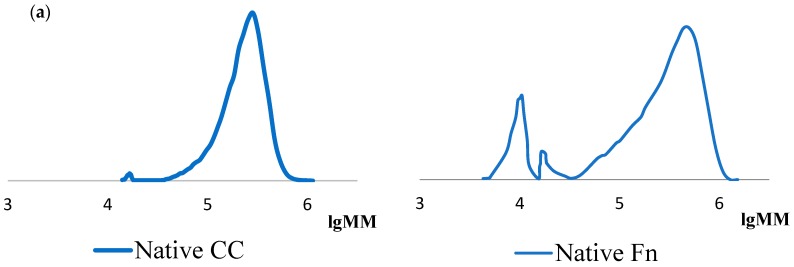

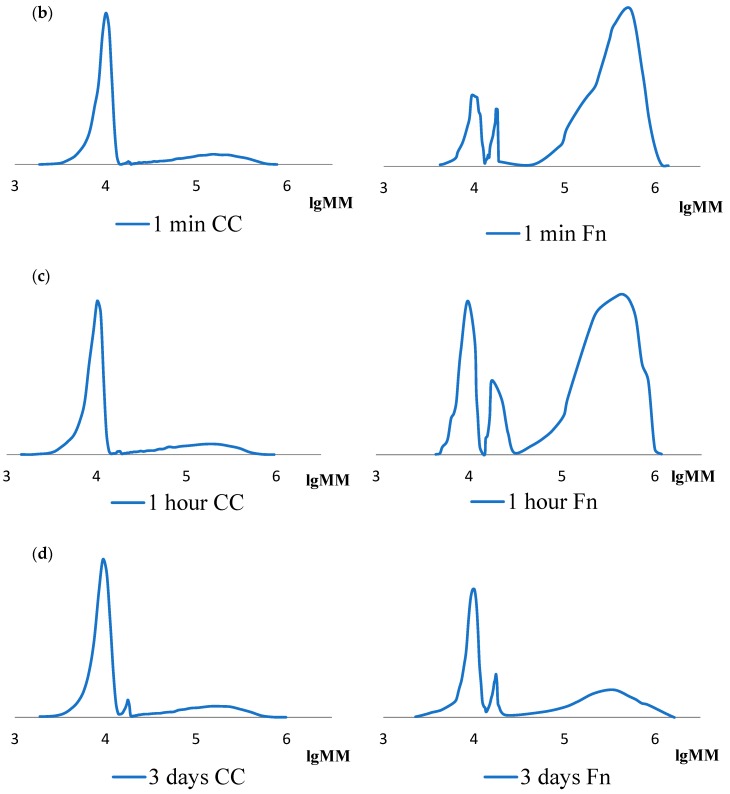
MMR curves of initial and hydrolyzed samples of cod collagen (CC) and fibrinogen (Fn) (ratio, protein: thrombin = 10^3^:1): (**a**) initial; (**b**) after 1 min; (**c**) after 1 h; (**d**) after 3 days of hydrolysis.

The same regularities were observed in the analysis of MMR curves of initial samples of cod collagen and fibrinogen, as well as their hydrolysates formed under the action of thrombin (Figure 1). It should also be noted that the polydispersity coefficient of low molecular fractions is 1.0–1.1. This indicates the homogeneity of the polymer in molecular weight values.

**Figure 2:**
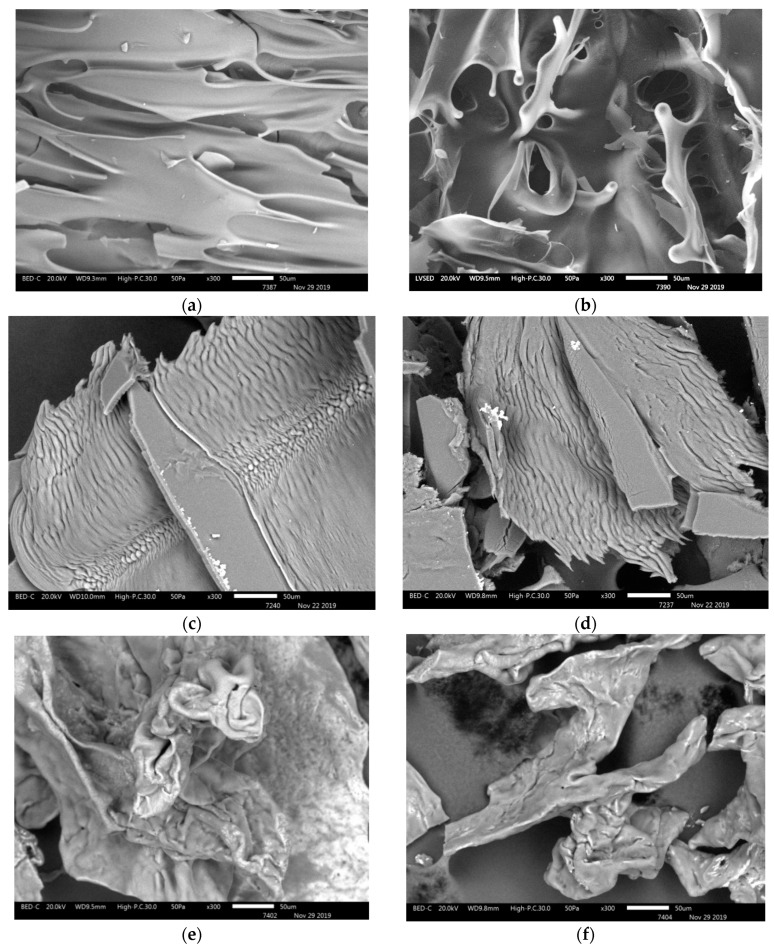
Microstructure of samples of initial fibrinogen (**a**,**b**), high molecular cod collagen (**c**,**d**) and fibrin-collagen scaffold (**e**,**f**).

The method of scanning electron microscopy was used in order to clarify the picture of the submolecular structure in the formation of scaffolds. Samples of the initial fibrinogen, high molecular collagen (sponge) and the scaffold based on them are presented in Figure 2.

It can be seen that fibrinogen does not have a fibrillar structure (Figure 2a,b), which is evident in the samples of collagen; closely spaced fibrils are visible (Figure 2c,d). Fragments of interconnected fibrils are visible in the structure of the scaffold surface; however, they are less extended than in the initial collagen (Figure 2e,f).

## 3. Discussion

These conclusions suggest a scaffold formation scheme based on fibrinogen and collagen. First, we should refer to the literature data, from which it is known that fibrin-polymer is formed from fibrinogen in three stages [30]. Two peptides A and two peptides B are cleaved from the fibrinogen molecule under the action of thrombin at the first stage, and then fibrin monomer is formed, built of two identical subunits connected by disulfide bonds. Each subunit consists of three different polypeptide chains. The fibrin monomer, when interacting with similar fibrin-monomers, forms a fibrin dimer at the second stage, then tetramer, octamer, etc., resulting in a large molecular complex, referred to as “soluble fibrin” or “soluble fibrin-monomer complexes”. Its M_w_ according to the literature data [30] is about 12,000–15,000 kDa. Fibrin-monomer aggregation (self-assembly of fibrin fibers) involves the transition of a molecule from a globule state to a fibril state. This fibrin is unstable and easily soluble in 5 M of urea acid or in 1% acetic acid. The fibrin-aggregate undergoes changes at the third stage; strong covalent bonds are formed between the polypeptide chains of fibrin-aggregate molecules, as a result of which it is stabilized into fibrin-polymer insoluble in concentrated urea solutions. In the case of collagen, enzymatic hydrolysis proceeds with low molecular fractions that can be identified and quantified [31].

Most likely, the scaffold formation during thrombin hydrolysis of fibrinogen and collagen mixture occurs in the following way. Hydrolysis of two proteins at room temperature is quite fast, especially collagen—almost a minute later, only oligomeric peptides with mainly M_w_ ~10 kDa are observed. Fibrinogen is destroyed more slowly; its destruction occurs according to the scheme described in the literature [30]. At the stage of fibrin-polymer formation, collagen hydrolysate fibers are embedded in the scaffold structure, which is accumulated in the system in a noticeable amount, forming the spatial structure of the scaffold.

## 4. Material and Methods

### 4.1. Preparation of Initial Materials for the Experiment

The following was used during the study:

- distilled water;
- solutions of acetic acid with a concentration of 3% and 4%, which were prepared from glacial acetic acid by diluting with distilled water;
- acetic acid solution of cod collagen with a concentration of 3%;
- fibrinogen (Fn)—commercial product extracted from human blood plasma (Sigma-Aldrich, Munich, Germany);
- enzyme—thrombin, proteolytic activity in 1 mL—50 IU (Research and Production Association «RENAM», Moscow, Russia);
- 1 M of sodium hydroxide solution, which was prepared from solid caustic soda by dissolving in a certain volume of distilled water;
- fibrin-collagen cell-free scaffold (provided by: Federal State Budgetary Educational Institution of Higher Education «Privolzhsky Research Medical University» of the Ministry of Health of the Russian Federation).

### 4.2. High Molecular Collagen Extraction Method

Collagen was extracted according to the author’s (copyright) method [32]. The covering tissues of the cod were cleaned, washed with cold tap water and then minced. The prepared raw material was weighed, washed first with cold tap water until the water was clean then 2 times with distilled water. Then the raw material was filled with 3% acetic acid solution at a liquid ratio (ratio of raw material and acetic acid solution) for cod—2. Solutions with covering cod tissue were left for 15 h with periodic stirring. The obtained collagen substance was filtered through filter paper. Collagen is stored at a temperature of 2–8 °C.

### 4.3. Methods of Protein Hydrolysis

Enzymatic hydrolysis was carried out at a temperature of 25 °C in an aqueous solution at pH ~7.0. Thrombin was used as an enzyme. The substrate–enzyme ratio in solution protein: thrombin 10^3^: 1.

The solution for hydrolysis of high molecular collagen was prepared by diluting the initial CC in acetic acid. Then 1 mol/L NaOH was added to the CC solution. In the case of Fn hydrolysis, its aqueous solution was prepared. Further, samples (1 mL) were taken in certain intervals after the addition of the enzyme: 1, 2, 3, 5, 8, 10, 20, 30 min, 1 h, 2 h, 3 days. To interrupt the hydrolysis, 1 mL of 4% acetic acid solution was added to the samples.

### 4.4. The Method of Analysis of Molecular Weight Characteristics

Molecular weight characteristics of hydrolysate of collagen and fibrinogen were determined by gel-penetrating chromatography (GPC) using a high-performance liquid chromatograph manufactured by Shimadzu CTO20A/20AC (Shimadzu, Kyoto, Japan) with the LC-Solutions-GPC software module. Separation was performed using columns Tosoh Bioscience TSKgel G3000SWxl (Tosoh, Tokyo, Japan) with a pore diameter of 5 microns. The low temperature light scattering detector ELSD-LT II (Shimadzu, Kyoto, Japan) was used as a detector. The 0.5 M acetic acid solution was used as eluent. The flow rate was 0.8 mL/min. Narrowly dispersed dextran samples with a molecular weight range (M_w_) of 1000–410,000 Da (Fluca) were used for calibration.

### 4.5. Method of Analysis of Collagen, Fibrinogen, Fibrin-Collagen Scaffold Structure

The research of the fibrin-collagen scaffold structure (provided by: Federal State Budgetary Educational Institution of Higher Education «Privolzhsky Research Medical Universit» of the Ministry of Health of the Russian Federation) and the initial proteins—high molecular weight cod collagen and fibrinogen, was carried out on the scanning electron microscope JSM-IT300 (JEOLL, Akishima, Japan). The scaffold sample was previously dissolved in a 3% solution of acetic acid and dried lyophilically. Samples of dehydrated proteins and scaffold were visualized. Dehydration of the samples took place directly in the chamber JSM-IT300 under the low vacuum impact.

## 5. Conclusions

The presented data allow three important conclusions to be drawn:

1. Low molecular fractions of collagen and fibrinogen hydrolysates have similar molecular weight values: M_w_ ~17–18 kDa and 10 kDa.
2. The destruction of macromolecules of high molecular collagen to hydrolysis products takes place almost completely within the first minute mainly to the polymer fraction with M_w_ ~10 kDa.
3. Enzymatic hydrolysis of fibrinogen goes slower than collagen, but it also occurs mainly to the polymer fraction with M_w_ ~10 kDa.

## Figures and Tables

**Table 1:** Molecular weight parameters of initial and thrombin hydrolyzed samples of high molecular cod collagen and fibrinogen (protein: thrombin = 10^3^:1).

Item No.	Sample	Parameter Values during Hydrolysis							
		Initial (M_w_, kDa)		1 min (M_w_, kDa)		60 min (M_w_, kDa)		3 days (M_w_, kDa)	
		Value	Fraction, %	Value	Fraction, %	Value	Fraction, %	Value	Fraction, %
1	CC	235–300	96	127	15	124	16	121	16
2		18	4	18	2	18	2	18	4
3		–	–	10	83	10	82	10	80
4	Fn	320–340	65	320–340	61	320–340	53	320–340	16
5		17	10	17	18	17	14	17	24
6		10	25	10	21	10	33	10	60
